# Cataract in HIV Patients: A Systematic Review and Meta-Analysis

**DOI:** 10.7759/cureus.72370

**Published:** 2024-10-25

**Authors:** Dillan Cunha Amaral, Lidia Cheidde, Bruno Fortaleza de Aquino Ferreira, Laura Cheidde, Pedro Paulo Ladeira Júnior, Isabelle Menezes, Vinícius Gomes, Bruno L. B. Esporcatte, Milton Ruiz Alves, Mário Luiz Ribeiro Monteiro, Joyce Hisae Yamamoto, Ricardo Noguera Louzada

**Affiliations:** 1 Ophthalmology, Federal University of Rio de Janeiro, Rio de Janeiro, BRA; 2 Faculty of Medicine, Pontifical Catholic University of São Paulo, Sorocaba, BRA; 3 Division of Ophthalmology and the Laboratory for Investigation in Ophthalmology (LIM-33), Faculty of Medicine, University of São Paulo, São Paulo, BRA; 4 Faculty of Medicine, City University of São Paulo, São Paulo, BRA; 5 Faculty of Medicine, State University of Rio Grande do Norte, Mossoró, BRA

**Keywords:** cataract, hiv, hiv aids, systematic review and meta-analysis, uveitis

## Abstract

HIV-induced AIDS attacks the immune system, leading to opportunistic infections. This syndrome has been linked to an increased risk of developing uveitis and subsequent cataracts. Consequently, cataract surgery may be associated with intra- and postoperative complications in HIV/AIDS patients. We conducted a systematic review and meta-analysis to investigate the impact of cataract surgery on individuals with HIV. The primary outcome of interest was the incidence of postoperative complications, including uveitis, as well as an analysis of potential risk factors. We systematically searched the PubMed, Embase, Web of Science, and Cochrane databases, identifying a total of 828 studies. Ultimately, four studies met our inclusion criteria. Of these, three studies exhibited a moderate risk of bias, while one study demonstrated a tendency toward a higher risk. Our analysis revealed that corrected distance visual acuity (CDVA) improved after cataract surgery, with a mean difference (MD) of -0.55 (-0.97; -0.12). This was derived from a sample characterized by heterogeneity (I² = 88%, τ² = 0.1429), with a p-value <0.01. Patients with a history of HIV-related uveitis showed less improvement in CDVA, with an MD of -0.30 (-1.03; 0.43). Regardless of the presence of prior uveitis, complications such as cystoid macular edema (CME), posterior capsular opacification, and postoperative uveitis were reported following cataract surgery. The overall prevalence of postoperative uveitis was estimated at 7% (95% CI: 1-13%) based on a random effects model, with heterogeneity measured at I² = 34%. Cataract surgery in HIV-positive patients results in significant improvements in visual acuity, although the presence of preoperative HIV-related uveitis may affect these outcomes. Postoperative complications, such as CME and uveitis, are more prevalent in this population and require careful management.

## Introduction and background

Cataracts are an ophthalmic condition characterized by lens opacity and a progressive decline in visual acuity [1]. They are more prevalent in women over the age of 50. Cataract surgery, which involves replacing the cloudy lens with an artificial one, is currently the most effective treatment available. Generally, the surgery has low complication rates in patients with normal health; however, this is not always the case for individuals with comorbidities such as HIV [2].

AIDS, caused by HIV, compromises the immune system by primarily targeting CD4+ T lymphocytes, rendering patients more susceptible to opportunistic infections and other health complications. Infection with HIV/AIDS has been linked to an increased risk of developing cataracts and a higher likelihood of requiring cataract surgery, particularly in individuals with low CD4 cell counts [3]. However, the literature regarding general postoperative complications in this population is inconsistent. Post-surgical outcomes for individuals with immunodeficiency can be particularly challenging, negatively affected by factors such as prior HIV-associated uveitis, a heightened risk of infections, and a slower healing process [4,5].

Although advancements in cataract surgical techniques and instrumentation have significantly reduced complications, making the procedure relatively safe for the general population, complications can still arise in HIV/AIDS patients [6].

Currently, meta-analyses addressing this topic are needed. Therefore, the purpose of our systematic review and meta-analysis is to consolidate and analyze all valid scientific evidence to understand the impact of cataract surgery on patients with HIV.

## Review

Methods

Our study was conducted and reported in accordance with the Cochrane Collaboration Handbook for Systematic Reviews of Interventions and the Preferred Reporting Items for Systematic Reviews and Meta-Analyses (PRISMA) Statement guidelines. The protocol was prospectively registered in the International Prospective Register of Systematic Reviews (PROSPERO) under the protocol number CRD42024557377.

Data Source and Search Strategy

We conducted a systematic search across PubMed, Embase, Web of Science, and Cochrane databases, with the last update on June 8, 2024. The complete search strategy included the following terms: (“HIV” OR “Human Immunodeficiency Virus” OR “AIDS” OR “Acquired Immunodeficiency Syndrome”) AND (“Cataract surgery” OR “Phacoemulsification” OR “phaco” OR “Manual Small-Incision Cataract Surgery” OR “MSICS” OR “MSCS” OR “Manual Small Incision Cataract Surgery” OR “SICS” OR “manual small-incision” OR “small incision cataract surgery”).

Two authors independently assessed all retrieved records, and any decisions regarding full-text retrieval were made through consensus. Full texts were then reviewed by the same two authors, who discussed the inclusion and exclusion criteria. Additionally, the references of eligible papers and systematic reviews were searched for further studies of interest.

Eligibility Criteria and Data Extraction

There were no restrictions regarding the publication date for study inclusion. We considered studies eligible if they reported on HIV patients undergoing cataract surgery, either phacoemulsification or manual small incision cataract surgery. However, conference abstracts and studies with overlapping patient populations were excluded.

Data on each of the specified outcomes were collected, along with the following baseline characteristics: (first author’s last name/year), study design, number of patients (eyes), mean age (years), number of males (%), status of highly active antiretroviral therapy (HAART) at the time of presentation, average CD4 count, other comorbidities associated with patients, intraocular pressure, and diagnoses (cataract or related). This data was extracted and recorded using an Excel template (Microsoft Corporation, Redmond, WA, USA) by two authors.

Outcomes of Interest

Our primary outcome of interest was the occurrence of postoperative complications, including uveitis and retinal detachment, along with the analysis of potential risk factors. Secondary outcomes included improvements in visual acuity, the need for surgical re-intervention, quality of life related to ocular health, and systemic complications.

Quality Assessment

Two independent authors evaluated the risk of bias in the included non-randomized controlled trials using the Cochrane tool for assessing risk of bias (ROBINS-I). Any disagreements were resolved through consensus. No randomized controlled trials meeting the inclusion criteria were identified.

Subgroup Analysis and Statistical Analysis

We conducted a subgroup analysis for patients with a history of previous uveitis. To evaluate efficacy and safety outcomes, a proportional meta-analysis was performed using the “metaprop” and “metacont” functions from the “meta” and “metafor” packages in R. Combined means and standard deviations were calculated using Cochrane’s formula, in accordance with recommended guidelines. Categorical endpoints were analyzed using RR with 95% CIs, while continuous outcomes were assessed with mean difference (MD). Heterogeneity was evaluated using I² statistics, with I² > 50% indicating substantial heterogeneity. For all endpoints, random effects models were applied. A p-value of < 0.05 was considered statistically significant. All statistical analyses were conducted using R software (version 4.2.3, R Foundation for Statistical Computing, Vienna, Austria).

Results

Study Selection

**Figure 1:**
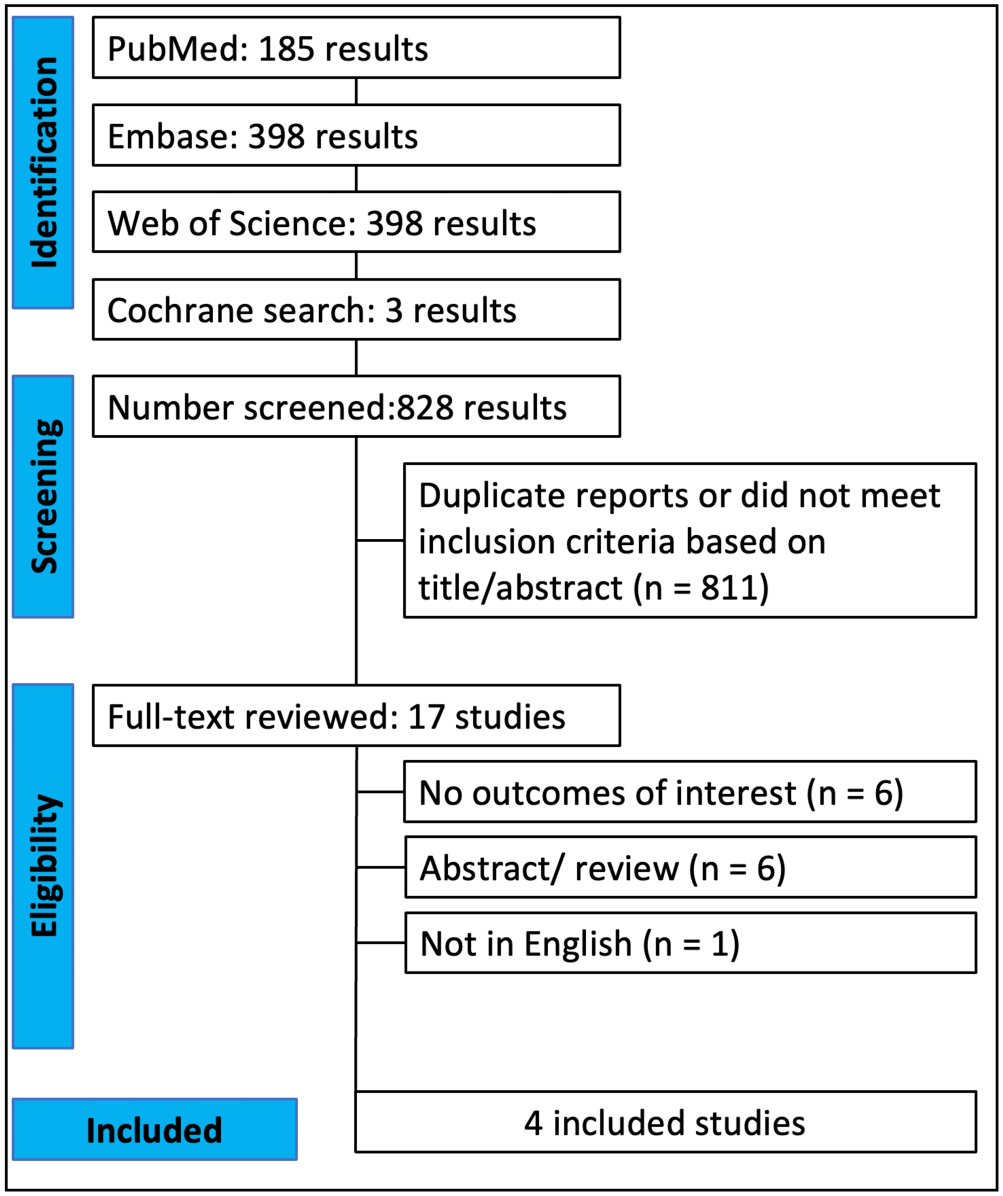
PRISMA flowchart of study selection PRISMA, Preferred Reporting Items for Systematic Reviews and Meta-Analyses

Figure 1 illustrates the study selection process. A total of 828 studies were identified: 398 from Embase, 185 from PubMed, 241 from the Web of Science, and three from Cochrane. Of these, 497 non-duplicate citations were screened, resulting in the exclusion of 480 based on title and abstract screening. The remaining 17 studies underwent a full-text review. Subsequently, 13 articles were removed due to incompatibility with the eligibility criteria: six did not report the outcomes of interest, six were of the wrong publication type, and one lacked a written or translated version in English. Ultimately, four studies were included, with no randomized controlled trials meeting the inclusion criteria identified.

Baseline Patients and Study Characteristics

Four retrospective studies were included in this meta-analysis, comprising two case-control studies [7,8] and two cohort studies [9,10]. Table 1 summarizes the baseline characteristics of HIV-positive patients undergoing cataract surgery across these studies.

**Table 1:** Baseline characteristics of included studies CMVR, cytomegalovirus retinitis; HAAT, highly active antiretroviral therapy; IRU, immune reactivation uveitis; NR, not reported; PSC, posterior subcapsular cataract

First author’s last name/year	Study design	Number of patients (eyes)	Mean age (years)	Number of males (%)	HAART at the time of presentation	Average CD4 count	Other comorbidities associated with patients	Intraocular pressure	Diagnoses (cataract or related)
Sankarananthan (2023) [7]	Retrospective case-control	107 (129)	52.2 +/- 8.9	55 males (51.4%)	93 patients (86.9%)	624 cells/cc (range: 73-1,670); CD4 count was available only in 43 patients	Eight patients had associated systemic tuberculosis	15.3 +/- 4.9 mmHg	Cataract: 40 (31%) mature cataracts, 88 (68.2%) immature cataracts, 1 (0.8%) traumatic cataract. OBS: Data for IMC and NS grade is available if necessary. Related: 1 (0.7%) – keratoconus, 1 (0.7%) – nebular corneal scar (herpes zoster sequelae), 2 (1.5%) – microcornea, 2 (1.5%) – primary angle glaucoma, 20 (15.5%) – HIV-related uveitis or retinitis
Accorinti (2019) (HIV+) [8]	Retrospective case-control	With uveitis before surgery: 11 (11); without uveitis before surgery: 21 (25)	With uveitis before surgery: 46.73 +/- 9.5 (33-63); without uveitis before surgery: 58 +/- 11.75 (37-74)	With uveitis before surgery: 6/11 (54.5%); without uveitis before surgery: 16/21 (76.19%)	NR	574.4 +/- 258.9 cell/mmc (range: 141-1,200); one month before surgery	With uveitis before surgery: comorbidities: 5 (45.45%), diabetes: 1 (9.1%), hypertension: 2 (18.18%), dyslipidemia: 2 (18.2%), HBV infection: 2 (18.2%), HCV infection: 3 (27.27%), pancreatitis: 1 (9.1%), TBC/Herpes Zoster: 1 (9.1%), heart lesions: 0, chronic renal failure: 0, thyroid changes: 0, lymphoma: 1 (9.1%), pulmonary lesions: 1 (9.1%), gastrointestinal lesions: 0, and pericarditis: 0. Without uveitis before surgery: comorbidities: 17 (80.95%), diabetes: 7 (33.3%), hypertension: 10 (47.6%), dyslipidemia: 11 (52.38%), HBV infection: 2 (9.52%), HCV infection: 1 (4.76%), pancreatitis: 1 (4.76%), TBC/Herpes Zoster: 2 (9.52%), heart lesions: 4 (19.04%), chronic renal failure: 1 (4.76%), thyroid changes: 2 (9.52%), lymphoma: 0, pulmonary lesions: 1 (4.76%), gastrointestinal lesions: 1 (4.76%), and pericarditis: 0	NR	With uveitis before surgery: bilateral cataract: 0, nuclear cataract (N): 0, cortical cataract (C): 0, PSC: 4 (36.36%), N+C: 0 C+S: 1 (9.1%), N+PSC: 4 (36.36%), and N+C+PSC: 1 (9.1%); total cataract: 1 (9.1%). Without uveitis before surgery: bilateral cataract: 4 (19.04%), nuclear cataract (N): 6 (24%), cortical cataract (C): 1 (4%), PSC: 2 (8%), N+C: 8 (32%), C+S: 0, N+PSC: 2 (8%), N+C+PSC: 4 (16%); total cataract: 2 (8%)
Miller (2021) (HIV+) [9]	Retrospective case-control	39 (66)	61.5 +/- 9	32 males (82.1%)	All patients	Mean of 697.3 cells/uL (SD = 335.7); range: 308-1,751	Type 2 diabetes: 7 (18%); heart disease: 9 (23.1%); treated chronic hypertension: 22 (56.4%); autoimmune disease: 3 (7.7%); age-related macular degeneration: 2 (5.1%)	NR	NR
Chew (2017) [10]	Retrospective cohort	29 (44)	55	27 males (93.10%)	86.2% of patients (n = 25)	266 cells/mm³ (range: 16-1,358 cells/mm³)	Six patients (10 eyes) had type 2 diabetes mellitus. The other eyes had mild or no diabetic retinopathy. Other comorbidities: pulmonary tuberculosis, hypercholesterolemia, hypertension, and ischemic heart disease	NR	Fourteen eyes (31.8%) had no ophthalmic manifestations of HIV/AIDS, while three eyes (6.8%) were diagnosed with CMVR. Additionally, 13 eyes (29.5%) had previously treated quiescent CMVR, five eyes (11.3%) presented with quiescent IRU, and two eyes had limbal stem cell deficiency with corneal scarring. Furthermore, two eyes had a history of gonococcal conjunctivitis, and one eye had a previous corneal abscess with corneal scar.

Sankarananthan et al. (2023) conducted a retrospective case-control study involving 107 patients (129 eyes) with retrovirus infection, of whom 32.8% had mature cataracts. Ninety-three patients (87%) were receiving HAART, with a mean CD4 count of 624 cells/mm³. Additionally, eight patients (7.5%) had associated systemic tuberculosis, and there were 129 eyes in the control group (HIV-negative) [7].

Accorinti et al. (2019) evaluated 32 HIV-positive patients (36 eyes), with a mean CD4 count of 574 cells/mm³. This retrospective case-control study compared 11 patients with uveitis/retinitis to 21 patients without, before surgery, demonstrating significant differences in comorbidities and types of cataracts between the groups, alongside a control group of 114 HIV-negative patients (166 eyes) [8].

Miller et al. (2021) performed a retrospective cohort study that evaluated 39 HIV-positive patients (66 eyes), with 56% having hypertension and 23% presenting with heart disease. All patients were on HAART, and the mean CD4 count was 697 cells/mm³, with a control group consisting of 5,949 HIV-negative patients (9,690 eyes) [9].

Chew et al. (2017) also conducted a retrospective cohort study, which included 29 HIV-positive patients (44 eyes), with 86% receiving HAART and a median CD4 count of 266 cells/mm³. Among these patients, six had type 2 diabetes mellitus [10].

Quality Assessment

**Figure 2:**
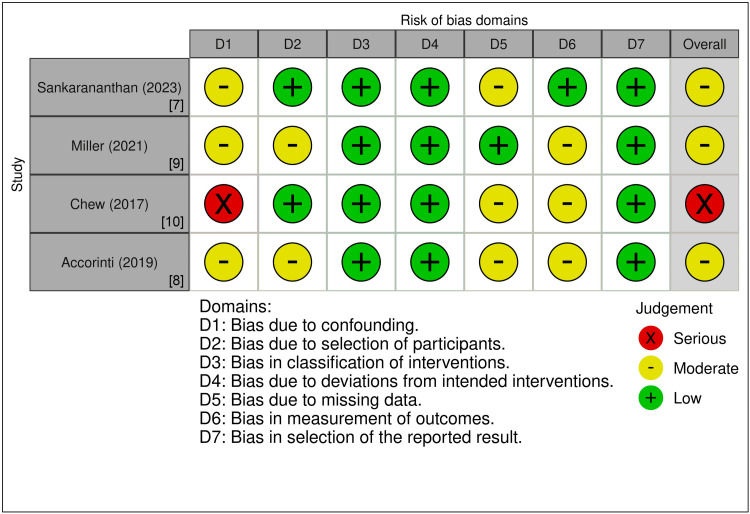
Quality assessment

Three of the studies were assessed to have a moderate overall risk of bias, as indicated by the analysis of the risk of bias domains using the ROBINS-I tool, as detailed in Figure 2. The study by Chew et al. (2017) exhibited a higher risk of bias, primarily due to the greater clinical complexity of the participants and the challenges in adequately controlling for multiple confounding factors (Figure 2) [10].

Pooled Analyses of All Studies

**Figure 3:**
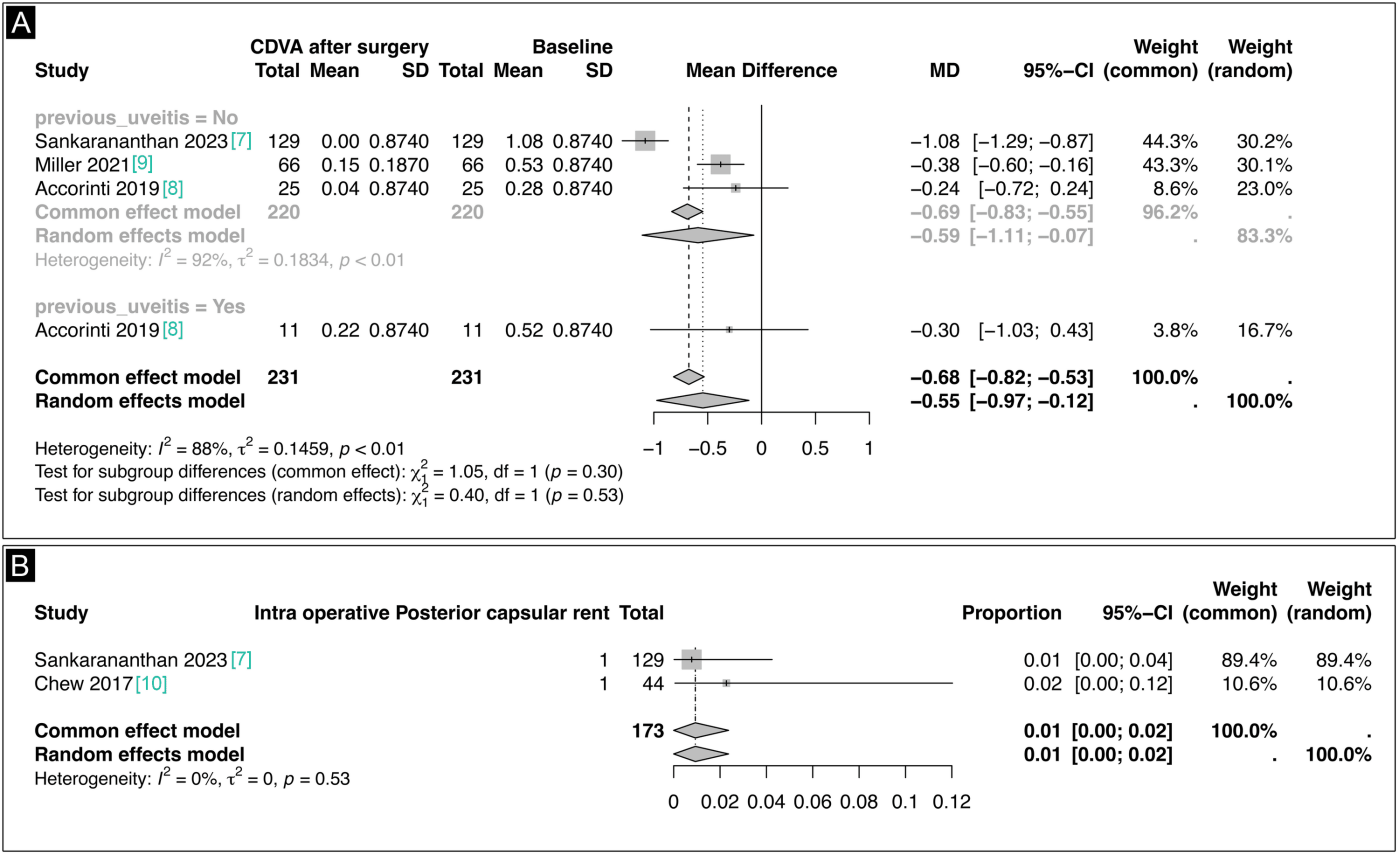
(A) Correct distance visual acuity forest plot. (B) Intraoperative posterior capsular rent forest plot

We analyzed the corrected distance visual acuity (CDVA) of HIV-positive patients before and after cataract surgery, stratified by the presence of prior uveitis (Figure 3A). The studies by Sankarananthan et al. (2023), Accorinti et al. (2019), and Miller et al. (2021) collectively reported outcomes for 231 eyes [7-9].

In the group without a history of uveitis, encompassing 220 eyes, we observed a significant improvement in CDVA post-surgery compared to baseline (MD -0.69; 95% CI -0.83 to -0.55; p < 0.001; I² = 92%; Figure 3A). This result was consistent in the random effects model (MD -0.59; 95% CI -1.11 to -0.07; p < 0.01; I² = 92%; Figure 3A). Accorinti et al.’s (2019) study included patients with prior uveitis, analyzing 11 eyes, which also demonstrated improvement in CDVA post-surgery (MD -0.30; 95% CI -1.03 to 0.43; Figure 3A) [8]. Pooling all studies revealed a notable overall improvement in CDVA post-surgery across both subgroups (MD -0.68; 95% CI -0.82 to -0.53; p < 0.001; I² = 88%; Figure 3A) in the common effect model, and (MD -0.55; 95% CI -0.97 to -0.12; p < 0.001; I² = 88%; Figure 3A) in the random effects model. No significant differences were found between patients with and without a history of uveitis (p = 0.30 for the common effect, p = 0.53 for random effects).

Regarding intraoperative complications, two studies reported the incidence of posterior capsular rent among 173 eyes. Sankarananthan et al. (2023) and Chew et al. (2017) reported proportions of 0.01 (95% CI 0.00-0.04) and 0.02 (95% CI 0.00-0.12), respectively [7,10]. The overall proportion of posterior capsular rent was 0.01 (95% CI 0.00-0.02; p = 0.53) in both the expected effect and random effects models (Figure 3B). There was no observed heterogeneity between studies (I² = 0%, p = 0.53), indicating a low and consistent occurrence of posterior capsular rent across studies.

**Figure 4:**
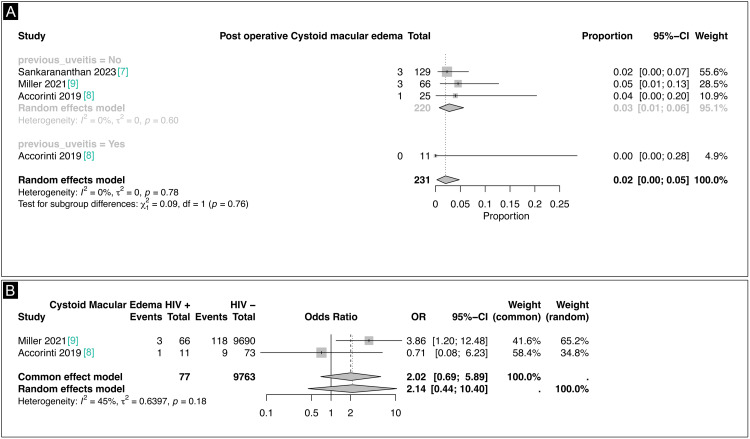
(A) Intraoperative CME forest plot. (B) CME in HIV + and in HIV – individuals forest plot CME, cystoid macular edema

Complications such as cystoid macular edema (CME), posterior capsular opacification (PCO), and uveitis were reported in the studies following cataract surgery. CME was specifically mentioned in the studies by Sankarananthan et al. (2023), Accorinti et al. (2019), and Miller et al. (2021) with treatment options including acetazolamide, topical non-steroidal anti-inflammatory drugs, and both topical and systemic steroids [7-9]. A total of 231 eyes were assessed, and patients were categorized based on the presence or absence of prior uveitis (Figure 4A). In patients without a history of uveitis, the combined proportion of CME occurrence was 0.03 (95% CI 0.01-0.06; I² = 0%, p = 0.60), with contributions from Sankarananthan et al. (2023), Accorinti et al. (2019), and Miller et al. (2021), indicating a low incidence of this complication [7-9]. Conversely, in patients with prior uveitis, the proportion was 0.0 (95% CI 0.00-0.28), based on data from Accorinti et al. (2019) [8]. There was no significant heterogeneity in either subgroup (I² = 0%, p = 0.78), and the test for subgroup differences showed no significant variation (χ² = 0.09, p = 0.76), suggesting that prior uveitis is not a major risk factor for CME.

We also analyzed the prevalence of CME in HIV-positive and HIV-negative patients. The studies by Miller et al. (2021) and Accorinti et al. (2019) included results for a total of 9,763 patients [8,9]. Miller et al. (2021) reported a higher incidence of CME in HIV-positive individuals (OR 3.86; 95% CI 1.20-12.48; p < 0.05; Figure 4B), while Accorinti et al. (2019) found no significant association between HIV status and CME (OR 0.71; 95% CI 0.08-6.23; p = 0.76; Figure 4B) [8,9]. When combining data from both studies, the common effect model yielded an overall OR of 2.02 (95% CI 0.69-5.89; p = 0.19; Figure 4B), while the random effects model showed an OR of 2.14 (95% CI 0.44-10.40; p = 0.33; Figure 4B). Both estimates suggest a trend toward a higher incidence of CME in HIV-positive patients, although the heterogeneity between studies was moderate (I² = 45%; p = 0.18).

**Figure 5:**
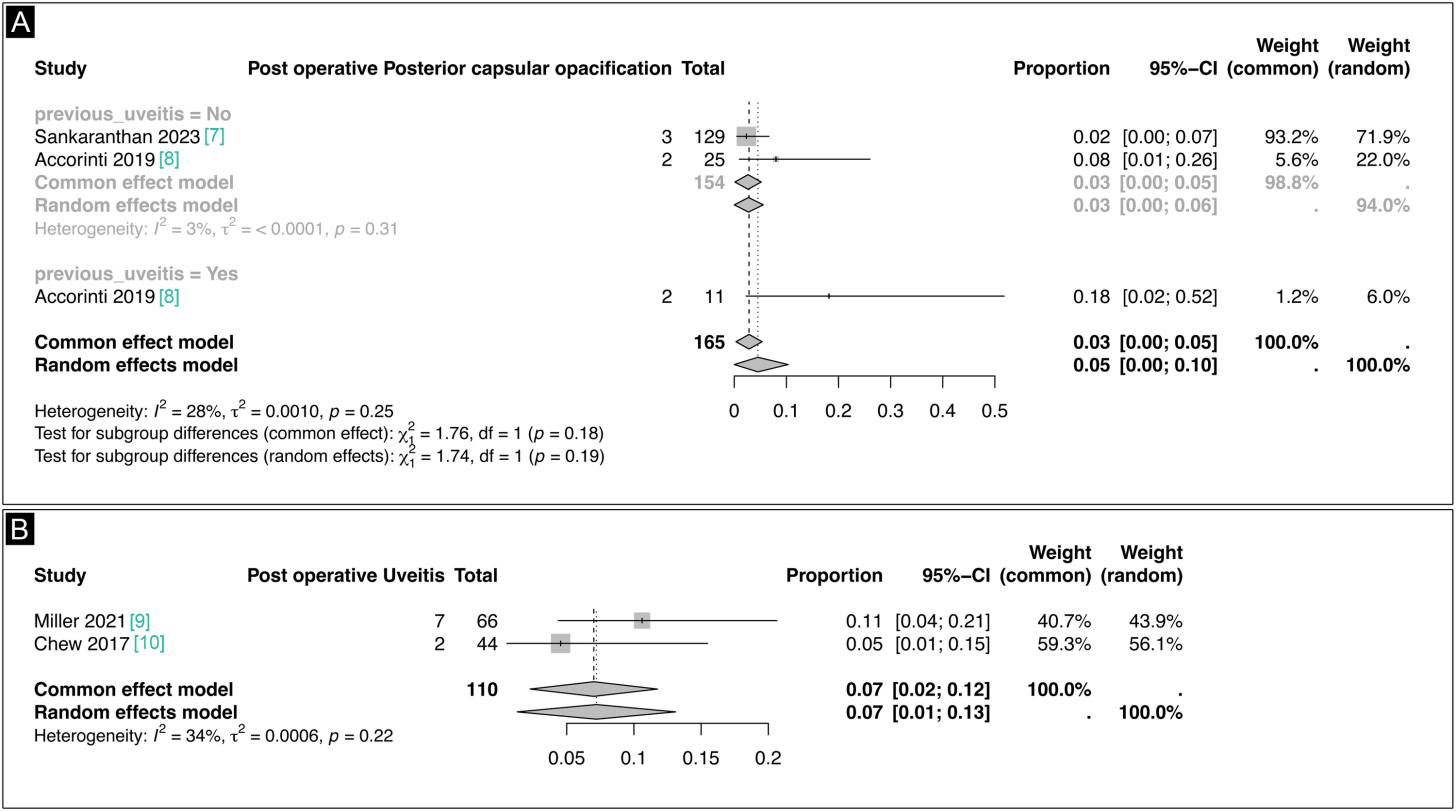
(A) Postoperative PCO forest plot. (B) Postoperative uveitis forest plot PCO, posterior capsular opacification

PCO was identified as a post-surgery complication, with Sankarananthan et al. (2023) and Accorinti et al. (2019) reporting on a total of 165 eyes [7,8]. Patients were categorized based on their history of uveitis. Among those without prior uveitis, the proportion of opacification was 0.03 (95% CI 0.00-0.05) in the common effect model and 0.03 (95% CI 0.00-0.06) in the random effects model (Figure 5A). The low heterogeneity (I² = 3%, p = 0.31) indicates a consistently reduced incidence in this subgroup. In contrast, for patients with prior uveitis, the common effects model showed a proportion of 0.03 (95% CI 0.00-0.05), while the random effects model indicated a proportion of 0.05 (95% CI 0.00-0.10) (Figure 5A). Despite this higher proportion, heterogeneity was moderate (I² = 28%, p = 0.25), indicating some variability. Tests for subgroup differences were not statistically significant (p = 0.18 and p = 0.19), suggesting that prior uveitis does not significantly influence the risk of PCO. Ultimately, although proportions were slightly higher in the uveitis group, no significant difference between subgroups was found, with both models demonstrating consistently low rates of opacification post-surgery.

Furthermore, the impact of uveitis on postoperative outcomes was described in the studies by Miller et al. (2021) and Chew et al. (2017) [9,10]. A total of 110 eyes were examined, revealing a proportion of 0.11 (95% CI 0.04-0.21; I² = 34%) in Miller et al.’s study and a proportion of 0.05 (95% CI 0.01-0.15; I² = 34%) in Chew et al.’s study (Figure 5B). The overall proportion for postoperative uveitis was 0.07 (95% CI 0.02-0.12) in the common effect model and 0.07 (95% CI 0.01-0.13) in the random effects model (Figure 5B). Low heterogeneity was observed (I² = 34%, p = 0.22), indicating consistency across the studies. These findings suggest a low but notable incidence of postoperative uveitis in the analyzed cohort.

Discussion

This systematic review and meta-analysis evaluated the outcomes and complications of cataract surgery in HIV-positive patients, providing valuable insights into the risks and benefits of this procedure within an immunocompromised population. A total of 6,377 patients were included, with 207 being HIV-positive and 6,170 HIV-negative. The results indicated that, despite complications, cataract surgery significantly improves visual acuity in HIV-positive patients.

The meta-analysis results revealed a significant enhancement in postoperative best CDVA across all studies, irrespective of prior uveitis. Patients without a history of uveitis exhibited marked improvement in CDVA, with substantial gains reported in the studies by Sankarananthan et al. (2023) and Miller et al. (2021) [7,9]. These findings align with existing literature identifying cataract surgery as effective in enhancing visual outcomes for HIV-positive patients [11]. However, Accorinti et al. (2019) noted that patients with prior uveitis experienced less improvement, suggesting that pre-existing ocular inflammation may adversely affect surgical outcomes [8]. The observed heterogeneity in CDVA improvement (I² = 89%) indicates variability in outcomes, likely attributable to differences in patient demographics, HIV management, and surgical techniques across studies.

Intraoperative complications, such as posterior capsule rupture, were rare but noted in the studies by Sankarananthan et al. (2023) and Chew et al. (2017) [8,10]. The low heterogeneity (I² = 0%) suggests a consistent risk across studies, with a combined incidence proportion of 0.01 (95% CI: 0.00; 0.02). This indicates that while the complication is infrequent, it remains a notable risk during cataract surgery in HIV-positive patients.

Postoperative complications, including CME, PCO, and uveitis, were reported across studies. CME was observed in both HIV-positive and HIV-negative populations, with Miller et al. (2021) indicating a higher prevalence in HIV-positive patients, while Accorinti et al. (2019) reported a higher prevalence in HIV-negative patients [8,9]. The lack of significant heterogeneity (I² = 45%) suggests a consistent risk of CME, with an overall higher incidence in the HIV-positive cohort; however, this result was not statistically significant (95% CI: 0.44; 10.40).

PCO was another complication analyzed, as reported by Sankarananthan et al. (2023) and Accorinti et al. (2019) [7,8]. The analysis revealed no significant difference in the occurrence of PCO between patients with and without prior uveitis (p = 0.19), indicating that a history of uveitis does not substantially impact the likelihood of developing PCO post-surgery.

Postoperative uveitis was evaluated using data from Miller et al. (2021) and Chew et al. (2017) [9,10]. The random effects model exhibited moderate heterogeneity (I² = 34%), suggesting variability in incidence among the included studies. The overall prevalence of postoperative uveitis was estimated at 7% (95% CI: 1-13%), indicating a significant risk of postoperative uveitis in HIV-positive patients.

In both treated and untreated HIV-positive individuals, impaired wound healing has been observed following more invasive surgeries. A declining CD4 count is associated with an increased risk of delayed wound healing and higher mortality rates in previous studies [11-13]. Notably, none of the patients in our analysis developed endophthalmitis, despite having low CD4 counts, suggesting that CD4 levels may not serve as a reliable predictor of postoperative infections. Furthermore, research on minor surgical wounds in HIV-positive patients indicated that CD4 counts did not influence the occurrence of wound infections [14]. This highlights the necessity for additional studies to elucidate the relationship between CD4 counts and complication risks. A comprehensive evaluation of the patient, accounting for both ocular and systemic comorbidities, is essential, with specific precautions taken when warranted. While HIV infection should be a consideration in surgical planning, it should not be the sole determinant in the decision-making process [15]. Although our findings did not demonstrate this relationship due to insufficient data, this meta-analysis underscores the importance of thorough preoperative assessment and careful postoperative management for HIV-positive patients undergoing cataract surgery. The increased risk of complications, such as CME and uveitis, necessitates vigilant monitoring and more aggressive anti-inflammatory treatment protocols [16]. Preoperative management should focus on achieving a quiescent eye for patients with a history of uveitis for an extended period before surgery [17]. While these patients can still benefit from cataract surgery, their postoperative outcomes may not be as favorable as those of patients without prior uveitis. Our data confirm that preoperative uveitis may attenuate visual gains, demonstrating less improvement in CDVA in these patients.

This meta-analysis has several limitations, including the retrospective nature of the included studies and the inherent biases associated with non-randomized designs. Small sample sizes and variability in reporting patterns among studies contribute to the observed heterogeneity and limit the generalizability of the findings. Additionally, the majority of studies reported data only for the HIV group, complicating statistical comparisons with the control group.

## Conclusions

Cataract surgery in HIV-positive patients typically results in significant improvements in visual acuity; however, the presence of preoperative uveitis may diminish these gains. Although the risk of intraoperative complications, such as posterior capsule rupture, is low, postoperative complications, including CME and uveitis, are more common and necessitate careful management. This meta-analysis emphasizes the importance of personalized surgical and postoperative strategies to optimize outcomes for HIV-positive individuals undergoing cataract surgery. Future research should prioritize prospective studies and randomized controlled trials to generate higher-quality evidence in this area.
